# Prevalence and clinical correlates of aggressive behavior in male inpatients with alcohol dependence during hospitalization

**DOI:** 10.3389/fpsyt.2026.1767212

**Published:** 2026-03-11

**Authors:** Yang Liu, Wenzheng Li, Haining Yu, Lina Gu, Yang Tian, Yu Liu, Dongmei Wang

**Affiliations:** 1 Affiliated Psychological Hospital of Anhui Medical University, Hefei, China; 2 Department of Addiction Medicine, Hefei Fourth People’s Hospital, Hefei, China; 3 Anhui Clinical Research Center for Mental Disorders, Anhui Mental Health Center, Hefei, China; 4 Institute of Psychology, Chinese Academy of Sciences, Beijing, China; 5 Department of Psychology, University of Chinese Academy of Sciences, Beijing, China

**Keywords:** aggressive behavior, alcohol dependent, clinical correlates, hemoglobin, hospitalization

## Abstract

**Objective:**

Aggressive behavior is a prevalent among hospitalized patients with alcohol dependence. However, its clinical correlates remain inadequately characterized. This cross-sectional study was designed to investigate the prevalence of aggressive behavior and identify associated clinical factors in this population during hospitalization, which is not well studied.

**Methods:**

Electronic medical records of 555 inpatients diagnosed with alcohol dependence were reviewed. Patients were stratified into aggressive (≥1 documented incident of overt interpersonal aggression during hospitalization) and non-aggressive behavior groups. Data collected included sociodemographics, suicide risk assessed using the Nurses’ Global Assessment of Suicide Risk (NGASR), and hematological parameters (blood routine test and hepatic function biomarkers).

**Results:**

The prevalence of aggressive behavior was 37.1% during the hospital stay in patients with alcohol dependence. In aggressive group, 45.1% demonstrated a single aggressive episode, 21.4% displayed two episodes and 33.5% experienced ≥3 episodes. Compared to the non-aggressive group, patients exhibiting aggression showed significantly shorter sleep duration, higher NGASR scores and decreased hemoglobin (all p<0.05). Logistic regression identified shorter sleep duration (p<0.001, OR = 0.749), higher NGSAR score (p = 0.004, OR = 1.146) and decreased hemoglobin (p = 0.003, OR = 0.981) as independent risk factors for aggressive behavior.

**Conclusion:**

Aggressive behavior is highly prevalent among hospitalized alcohol-dependent patients. Shorter sleep duration, higher suicide risk and decreased hemoglobin are significant clinical and hematological correlates of this behavior during hospitalization. These factors warrant clinical attention for risk assessment and management.

## Introduction

Alcohol use disorder (AUD), affecting over 283 million people worldwide (1), arises from chronic excessive alcohol consumption and confers substantial societal burdens. AUD is strongly associated with adverse outcomes including injuries, violence perpetration, sexual victimization, cognitive impairment, and premature mortality (2). Aggressive behaviors—particularly impulsive aggression (3), manifest in 40-78% of patients with AUD (4–8). A study carried out in a male psychiatric ward of a general hospital found that alcohol withdrawal was the leading factor behind aggressive incidents, representing 45.7% of cases. This was followed by schizophrenia and mania, which together accounted for 43.0% of violent events in the unit (9). A survey found that a shocking 91.5% of psychiatric service workers have experienced some kind of aggression or injury on the job (10). Aggressive behavior that occurs during hospitalization profoundly impact patients’ families (5), healthcare systems (11–13), psychiatric staff (10, 14), and critically impair patients’ treatment adherence and long-term recovery (15).

Impulsive aggression—characterized by diminished inhibitory control and heightened reactivity to perceived threats—represents the most clinically relevant phenotype in AUD (16). Previous studies have found that impulsive trait is not only one of a key factor leading to alcohol dependence, but also an important factor of aggressive behavior in patients with alcohol dependence (17, 18). This pathological manifestation features a lowered threshold for aggressive motor responses to provocation, with reduced consideration of behavioral consequences (16). AUD patients exhibit amplified impulsive aggression due to alcohol’s neuropharmacological disruption of prefrontal-amygdala circuitry (19), demonstrating pronounced negative urgency during emotional distress that escalates aggression risk during hospitalization.

To identify additional strategies for more effectively preventing and managing aggression among patients with alcohol dependence, a growing number of studies worldwide have begun to investigate its associated factors. These studies have found several factors that could be linked aggressive behavior in individuals with alcohol dependence, spanning biological, sociological, and psychological domains. For example, a study from Switzerland found that being male and younger in age can be linked to an increased likelihood of displaying violent behavior when under the influence of alcohol (20). Some studies have found that heightened aggression correlates with both non-fatal suicide attempts (21) and completed suicide (22, 23). In addition, the severity of alcohol withdrawal significantly increases the risk of both aggression and suicide (24). Research on impulsive aggression within psychiatric inpatient settings has indicated that several patient-related risk factors are closely linked to incidents of violence during hospitalization. These include a history of previous aggressive behavior, extended length of stay, involuntary admission, tendencies toward impulsivity and hostility, limited awareness of one’s condition, and cases where both the aggressor and the victim share the same gender (25, 26). Hemoglobin (Hb) is a key protein that carries oxygen in red blood cells. Changes in Hb content directly affect the oxygen supply to the brain tissue, leading to the decline of cognitive function, which may reduce the judgment ability of patients and increase the risk of violence (27). Studies have found that anemia can lead to anxiety and depression and other psychiatric symptoms, thereby increasing the risk of violence (28, 29). A study on 50,311 children found that those diagnosed with anemia had greater irritability than children not diagnosed with anemia (30). In Alzheimer’s disease, decreased Hb is associated with behavioral symptoms such as agitation and disinhibition (31). Therefore, early identification of potential risk factors for aggressive behavior is crucial for prevention of aggression in alcohol dependent patients.

To our best knowledge, this is the first study focusing on the incidence of aggressive behavior in alcohol-dependent patients during hospitalization. The aims of the present study was undertaken to estimate the prevalence of aggressive behavior among alcohol dependent patients during hospitalization and to explore sociodemographic, clinical symptoms and common clinical biological indicators (such as Hb) that may be related to the aggressive behavior.

## Methods

### Participants

A total of 555 consecutive male inpatients diagnosed with alcohol dependence according to ICD-10 criteria (32) were retrospectively identified through the electronic medical record system of Hefei Fourth People’s Hospital between January 2022 and September 2024. Our institution - Hefei Fourth People’s Hospital - is located in Hefei City, Anhui Province, China. As of the end of 2024, the permanent population of Anhui Province was 61.23 million. Hefei Fourth People’s Hospital is a tertiary grade A mental health specialized hospital with 1,633 beds. The annual outpatient volume exceeds 500,000 visits. The main diseases treated include schizophrenia, emotional disorders, alcohol addiction, etc”.

Inclusion criteria comprised: (1) Age between 18 and 70 years; (2) Alcohol dependence is the primary clinical diagnosis; (3) Capacity to provide informed consent; (4) Ability to complete assessments independently or with researcher assistance.

The mean age of patients was 46.25± 10.40 years, with a mean length of stay of 35.71± 25.57 days. Participants were stratified into aggressive (n = 206) and non-aggressive (n = 349) groups based on documented evidence of ≥1 overt interpersonal aggression incident during hospitalization, operationalized as physical or verbal acts causing harm to others or property (16). During hospitalization, patients received supervised pharmacotherapy (diazepam substitution therapy and other symptomatic treatments)with complete alcohol abstinence and controlled smoking schedules.

Exclusion criteria were: (1) Patients with serious illnesses (such as infectious, cancer, immune system diseases etc.) that could significantly confound the results of the study other than anemia; (2) History of substance abuse other than alcohol and tobacco; (3) Serious neurological diseases other than those caused by alcoholism; (4) Patients with a prior diagnosis of severe mental disorder (ICD-10 criteria) confirmed through systematic electronic medical record review, such as manic episode, schizophrenia, etc; (5) Declined participation.

This research received ethical clearance from the Ethics Committee of the Hefei Fourth People’s Hospital (HFSY-IRB-YJ-KYXM-LY 2024-066-001), adhering to the principles outlined in the Helsinki Declaration. Additionally, the project was formally approved by the Anhui Medical University. All participants were thoroughly informed regarding the objectives and specifics of the research. All participants (or their legal guardians) provided written informed consent for the publication of this study. The manuscript contains no personally identifiable information, such as names, dates of birth, or hospital record numbers.

### Sociodemographic characteristics and clinical variables

Demographic and clinical characteristics were collected through the electronic medical record system: Sociodemographics including age, marital status (categorized), education years, body mass index (BMI) and smoking history. Clinical variables including: age of first use (age at first regular drinking), years of alcohol use (the period from the first of regular drinking to this hospital admission), Quantity of use per day (Daily alcohol consumption was converted to pure alcohol intake) and Length of stay.

### Clinical symptoms

The severity of alcohol withdrawal was assessed using the CIWA-Ar (Clinic Institute Alcohol Withdrawal Syndrome Scale) (33). The CIWA-Ar scale is a tool explicitly recommended by ASAM (American Society of Addiction Medicine) in evaluation of the severity of alcohol withdrawal (34). Its clinical application includes the recording of the maximum daily score to reflect the peak severity of the patient’s withdrawal symptoms (33). Competent nurses can carry out an evaluation in less than two minutes and the inter-rater reliability is high (r>0.8) (35). The CIWA-Ar (Chinese version) has been shown to have good reliability and validity (α=0.85) in Chinese populations (36)”. Based on the severity of withdrawal symptoms, the CIWA-Ar was evaluated at least once a day in our study. Since alcohol withdrawal symptoms usually disappear within one week with timely treatment, and the course of delirium is generally no more than 15 days, the scale is evaluated for a total of two weeks. Higher CIWA-Ar scores indicate greater severity of alcohol withdrawal. In this study, the maximum CIWA-Ar scores of the patients after admission was selected.

The Nurses’ Global Assessment of Suicide Risk (NGASR) was administered to evaluate suicide risk (37). This evidence-based instrument incorporates 15 clinically validated indicators of suicide risk, with each item scored dichotomously (0 = absent, 1 = present) to yield a total score range of 0-15. Higher total scores indicate greater suicide risk severity. The Chinese version demonstreated good reliability and convergent validity when cross-validated against the Beck Hopelessness Scale (BSS) and Beck Scale for Suicide Ideation (BSI) (38).

Sleep duration was evaluated through the validated query: *“During the past 30 days, how many hours of actual sleep did you typically obtain per night?”) (*39, 40). In this study, the sleep duration was inquired by nurses on the first day of admission.

### Measurement of blood parameters

Venous blood samples were collected from all participants between 6:00 and 7:00 am within 24 hours of hospital admission. Samples underwent immediate cryopreservation and were transported on dry ice to the institutional clinical laboratory. Blood routine test including white blood cells (WBC), Hb, and platelets (PLT) were quantified using an automated hematology analyzer by hospital laboratory technicians. Hepatic biomarkers including total bilirubin (TBIL), direct bilirubin (DBIL), alanine aminotransferase (ALT), aspartate aminotransferase (AST), gamma-glutamyl transpeptidase (GGT) simultaneously analyzed with an automated biochemistry platform (Roche Cobas 8000).

### Statistical analysis

Statistical analyses were performed using SPSS 26.0 (IBM, Chicago, IL, USA) The distribution of the sample was examined using the Kolmogorov-Smirnov one-sample test. Between-group comparisons employed χ² tests for categorical variables and one-way ANOVA for continuous variables. Analysis of covariance (ANCOVA) was subsequently employed to compare NGSAR scores, sleep duration and blood routine indexes between aggressive and non-aggressive groups. incorporating significant demographic and clinical correlates as covariates.

Stepwise backward logistic regression (Wald method) were used to identify independent factors of aggressive behavior in alcohol dependent patients during hospitalization. Significance was defined as two-tailed α = 0.05, with Bonferroni correction applied to all primary analyses to address multiple comparisons. Model performance was evaluated with explained variance (Nagelkerke’s R²), calibration (Hosmer-Lemeshow test).

## Results

### Prevalence and frequency distribution of aggressive episodes during hospitalization in alcohol-dependent patient

**Figure 1:**
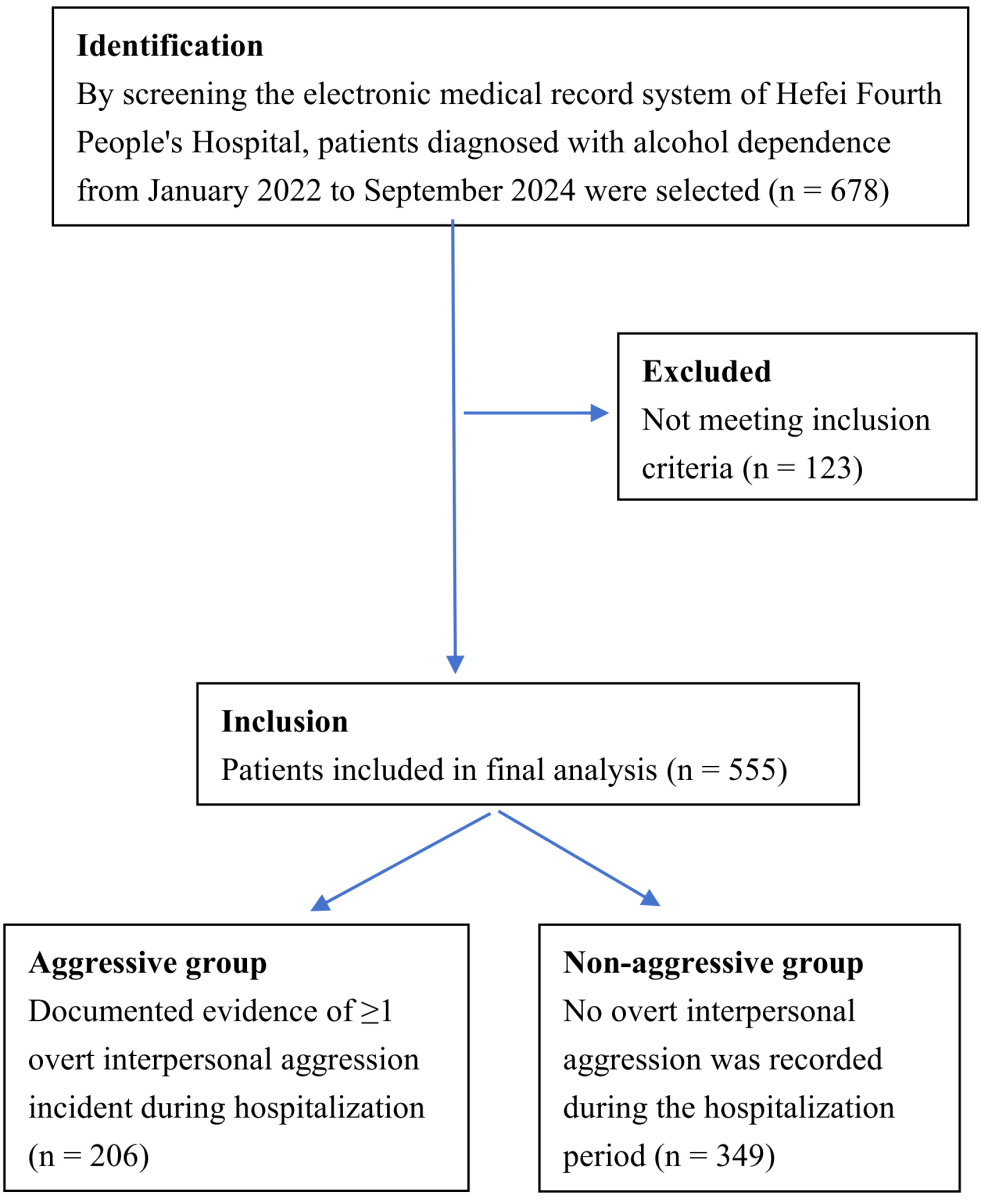
Flow diagram of patient selection and grouping.

The patient selection flowchart and grouping are shown in **Figure 1**. Among the 555 selected hospitalized alcohol-dependent patients, 206 individuals (37.1%) exhibited aggressive behavior during admission. The incident frequency distribution revealed that 93 patients (16.8% of total cohort; 45.1% of aggressive subgroup) demonstrated a single episode, 44 (7.9%; 21.4%) displayed two episodes, and 69 (12.4%; 33.5%) experienced ≥3 aggressive episodes.

### Clinical characteristics and hematological indices in alcohol dependent patients with aggressive behavior vs those without aggressive behavior

Comparative analysis of 206 aggressive and 349 non-aggressive alcohol-dependent inpatients revealed no significant group differences in age, education duration, marital status, smoking history, age of first use, years of alcohol use, quantity of use per day and length of stay (all p > 0.05). Significant differences existed between two groups in CIWA-Ar score (p=0.001). This significant variable was adjusted for in subsequent statistical analyses as covariate.

Patients with aggressive behavior demonstrated clinically significant alterations: reduced sleep duration (Adjusted p<0.001), elevated NGARS-assessed suicide risk (Adjusted p= 0.006) and decreased Hb concentration (Adjusted p=0.002) compared to the non-aggressive group. Following Bonferroni correction, decreased Hb and sleep reduction retained statistical significance (**Table 1**).

**Table 1:** Socio-demographic as well as blood routine and biochemical parameters featuring in alcohol dependent inpatients with aggressive and without aggressive behavior.

Variables	Without Aggressive behavior (n=349)	With aggressive behavior (n=206)	F/X^2^	P-value	Adjusted p’-value
Age, years	46.76 ± 10.26	45.43 ± 10.59	2.154	0.143	
Education, years	9.55 ± 3.35	9.32 ± 3.47	2.224	0.136	
BMI, kg/m2	22.71 ± 3.60	22.21 ± 3.54	2.224	0.136	
Marital status:			5.287	0.071	
Single, n(%)	36(10.3%)	33(15.9%)			
Married, n(%)	239(68.5%)	124(59.9%)			
Divorced/Widowed, n(%)	74(21.2)	50(21.2%)			
Smoking history:			1.569	0.210	
Never smoker	41(11.7%)	32(15.5%)			
Current smoker	308(88.3%)	175(84.5%)			
Age of first use, years	25.19 ± 8.28	25.35 ± 9.02	0.048	0.827	
Quantity of use per day (g/per day)	217.08 ± 221.66	221.66 ± 94.69	0.050	0.824	
Years of alcohol use, years	22.08± 10.01	20.47± 10.62	3.280	0.071	
Length of stay, days	33.49 ± 24.15	37.84 ± 27.61	3.56	0.060	
CIWA-Ar	9.04 ± 6.44	11.00 ± 7.08	11.11*	0.001*	
NGASR	2.38 ± 1.66	2.93 ± 2.38	10.100	0.002*	0.006*
Sleep duration, hours	5.38 ± 1.69	4.55 ± 1.89	28.279	<0.001*	<0.001*
Hepatic function biomarkers					
TBIL, μmol/L	18.21 ± 13.11	17.55 ± 12.39	0.311	0.565	0.532
DBI, μmol/L	7.78 ± 6.51	7.69 ± 6.79	0.019	0.890	0.879
ALT, U/L	42.30 ± 47.36	36.97 ± 35.39	1.887	0.170	0.303
GGT, U/L	240.84 ± 396.93	241.22 ± 397.98	0.000	0.987	0.877
AST, U/L	62.00 ± 70.55	58.86 ± 56.40	0.285	0.594	0.864
Blood routine					
Hb, g/L	137.73 ± 17.20	130.05 ± 15.81	21.32	0.000*	0.002*
WBC, 10^9^/L	6.78 ± 2.17	6.92 ± 2.05	7.714	0.556	0.456

Values are presented as mean ± SD; *statistically significant at p<0.05 level. Adjusted p’-value: The p values for NGASR,Sleep duration,Hepatic function biomarkers Blood routine.

adjusted for CIWA-Ar score as covariates. Abbreviations: BMI, body mass index; NGASR, nurses global assessment of suicide risk; CIWA-Ar, Clinic Institute Alcohol Withdrawal Syndrome Scale; TBIL, total bilirubin; DBIL, direct bilirubin; ALT, alanine aminotransferase; AST, aspartate aminotransferase; GGT, gamma-glutamyl transpeptidase; WBC, white blood cells; Hb, hemoglobin.

### Logistic regression

We then focused on the risk factors of aggressive behavior in alcohol dependent patients during hospitalization. The significantly different variables of univariate analysis were included in logistic regression (Backward: Wald) to detect the risk factors of aggressive behavior in alcohol dependent patients. As shown in **Table 2**, the risk factors of aggressive behavior in alcohol dependent patients during hospitalization were as follows: reduced sleep duration (p<0.001, OR = 0.749), elevated NGARS-assessed suicide risk (p = 0.004, OR = 1.146), and decreased Hb concentration (p = 0.003, OR = 0.981). The Hosmer-Lemeshow test yielded a P value of 0.455. Nagelkerke R² = 0.116.

**Table 2:** Logistic regression models of aggressive behavior in patients with alcohol dependence.

Predictor	OR (95%Cl)	P value
Sleep duration	0.749(0.671-0.836)	0.000*
NGSAR	1.146(1.037-1.253)	0.004*
Hb	0.981(0.969-0.994)	0.003*

*statistically significant at p<0.05 level; CI, confidence interval;.

OR, odds ratio; NGASR, nurse’s global assessment of suicide risk; Hb, hemoglobin;.

Hosmer-Lemeshow (p=0.455). Nagelkerke R² = 0.116.

## Discussion

This represents, to the best of our knowledge, the first large-scale investigation documenting a 37.1% prevalence of inpatient aggressive behaviors among alcohol-dependent patients, with analysis revealing three significant clinical correlates: reduced sleep duration, elevated NGASR scores, and decreased Hb concentrations. Notably, decreased Hb demonstrated significant associations with aggression, establishing hematological biomarker as novel predictors of behavioral dysregulation during alcohol withdrawal hospitalization.

### Prevalence of aggressive behavior in male inpatients with alcohol dependence during hospitalization

Our results indicated that the prevalence of aggressive behavior in male inpatients was 37.1%. A six-month study of aggressive behavior in 56 patients with alcohol dependence found that about 89.28% eliciting aggression on trivial matters which did not agitate them usually (41). A study on risk factor for violent offending showed that 72% of the patients with an alcohol use disorder had committed a violent crime leading to admission (42). Our study focused on prevalence of aggressive behavior during hospitalization in alcohol-dependent patients rather than a history of aggressive behavior. To date, there have been no reports on the incidence of aggressive behavior in alcohol-dependent patients during hospitalization. Our study revealed that the prevalence of aggressive behavior during hospitalization was significantly higher among alcohol-dependent patients than the reported incidence rates among general psychiatric inpatients. This finding aligns with prior research, including a study conducted at a tertiary mental health facility in India, which reported that 8.52% of individuals admitted to an acute psychiatric unit experienced at least one episode of violence during their hospitalization (26). Earlier Western studies have also indicated that between 3% and 44% of patients in psychiatric wards engaged in violent behavior during their stay, with higher prevalence rates observed in inpatient units characterized by a larger proportion of male patients, involuntary admissions, and individuals with alcohol dependence (43). The elevated incidence of aggressive behavior in alcohol-dependent patients may stem from heightened impulsivity associated with neurobiological alterations, as neuroimaging studies reveal structural and functional abnormalities in impulse-regulation circuits. Chronic alcohol exposure induces frontal cortical-striatal circuitry dysregulation, enhancing impulsive behavior and weakening inhibitory control (44), while concurrent reductions in striatal grey matter volumes compromise neural substrates essential for behavioral inhibition (45).

### Clinical symptoms and aggressive behavior in male inpatients with alcohol dependence during hospitalization

In this study, we found that aggressive behavior was associated with shorter sleep duration in alcohol dependent patients that is consistent with the results of other studies in different populations (46, 47). Short sleep duration is not only significantly positively correlated with aggressive behavior, but also may promote or aggravate aggressive behavior by affecting emotional regulation, behavior control and other mechanisms” (48, 49). Cheng et al (50) explored the connections between sleep patterns, brain anatomy, and cognitive as well as psychiatric problems, proposing that disrupted sleep might worsen abnormalities in brain function, which in turn can affect impulse control. Therefore, the increased aggression caused by sleep disorders may be due to the fact that sleep disorders reduce impulse control or self-control. However, it should be pointed out that our study can only illustrate the correlation between sleep duration and aggressive behavior in hospitalized patients with alcohol dependence, and cannot conclude the correlation between sleep quality and aggressive behavior in patients with alcohol dependence.

Alcohol consumption constitutes a significant population-level risk factor for suicide (51, 52). Studies comparing alcohol-dependent individuals with and without suicide attempts indicate substantially more severe addiction profiles among attempters (53). Our findings demonstrate a significant association between aggressive behavior and NGASR scores in patients with alcohol dependence. This aligns with established literature linking both aggression and suicidality in psychiatric disorders (22, 23, 54, 55) and specifically in alcohol dependence (56, 57). Although aggression and suicide represent distinct psychopathological manifestations—with suicidal behavior conceptualized as inwardly-directed violence—they share fundamental neurobiological substrates. Convergent evidence implicates dysregulation in central serotonergic (5-HT) neurotransmission as a common mechanism underlying aggression, depression, and suicidal behavior (58).

Aggressive behavior in alcohol-dependent patients may further stem from psychological sequelae of alcohol withdrawal syndrome (AWS), where symptoms including anxiety, depression, and restlessness diminish behavioral control, thereby elevating aggression risk. Because the severity of alcohol withdrawal symptoms was a well-known and important risk factor for aggressive behavior, we controlled for the severity of alcohol withdrawal as a covariate in this study.

### Aggressive behavior and Hb in patients with alcohol dependence

Our study found that decreased Hb concentration was associated with aggressive behavior in patients with alcohol dependence, which is consistent with the results of some previous studies (59, 60). Lower Hb levels are associated with poorer cognitive function, and cognitive deficits may further lead to impulsive behavior (27). Lower Hb levels are also associated with an increased risk of psychological disorders, such as anxiety and depression, which often accompany impulsive behaviour (61). Mechanistically, Hb is a key protein that carries oxygen in red blood cells, and its content changes directly affect the oxygen supply of brain tissue. Anemia leads to a decrease in Hb content, which causes brain hypoxia and affects the function of neurons in key regions of the brain such as the prefrontal lobe and the connectivity of brain regions. Thus, it increases the risk of individual violent behaviors (27). Lower Hb may indirectly regulate the dopamine and serotonin systems, which play a key role in impulsive behavior, by affecting oxygen supply and neurometabolism (62, 63). In addition, the severity of alcohol consumption and withdrawal are important risk factors for aggressive behavior. The mechanism may also involve changes in Hb. The severity of alcohol consumption is associated with lower Hb levels, and alcohol increases the risk of anemia such as iron deficiency anemia (64) and B12-deficiency anemia (65). Alcohol may also directly cause hemolysis by inducing increased red blood cell (RBC) fragility and eryptosis, which abates after a week of abstinence (66). Meanwhile, the high inflammatory state during the withdrawal period may further affect Hb stability through oxidative stress (66, 67). Thus, Hb may be considered as a potential proxy for the severity of alcohol consumption and withdrawal. It is worth noting that previous studies mostly focused on the association between mild or severe anemia and psychiatric symptoms, but in our study, the two groups of patients did not meet the diagnostic criteria for anemia (Hb < 130g) (68). This phenomenon might be attributed to the fact that long - term alcohol consumption leads to extensive damage to the nervous system via multiple pathways (e.g., neurotoxicity, inflammation, oxidative stress, autonomic nervous disorders). The nervous system’s sensitivity to the reduction of Hb is notably heightened, and even a minor decline in Hb content can trigger mental abnormalities, which may result in agressive behavior. Therefore, the decrease in Hb among patients with alcohol dependence could essentially serve as a marker of systemic multisystem damage, and the emotional instability it triggers reflects the overall decompensation of the neurovascular unit under the condition of chronic alcoholism. However, higher Hb can also lead to impaired cerebral oxygenation and perfusion affecting cognitive function (69) and secondary aggressive behavior. Hb levels demonstrate a complex bidirectional association with impulsive behavior in alcohol-dependent patients, potentially influencing aggression risk through multiple pathways. Lower Hb levels (anemia) may impair impulse control by inhibiting prefrontal cortex activity - a key regulatory region (59) while elevated Hb concentrations could exacerbate impulsivity through oxygenation and perfusion mechanisms (69, 70). This suggests neurobiological interactions involving inflammation, neuroendocrine signaling, and cerebral oxygenation may underlie Hb-aggression relationships (71–73). Though precise mechanisms require further investigation, clinically, optimizing Hb levels may represent a novel therapeutic strategy for aggression management in this population.

This study has several limitations. First, the case-control design precludes causal inferences between identified factors and aggressive behavior in alcohol-dependent inpatients; prospective cohort studies are needed for validation. Secondly, participants were exclusively Han Chinese males recruited from inpatient settings, necessitating validation in ethnically diverse populations across clinical contexts. Third, in this study, subjects received benzodiazepines based on the severity of alcohol withdrawal symptoms after admission. However, specific medication types and dosages were not included as variables in the analysis. Incorporating pharmacotherapy information would be of significant clinical importance for the conclusions of this study. In future research, we will systematically collect detailed treatment information to further validate and extend our findings. Fourth, in this study, the highest score of CIWA-AR scale could not comprehensively reveal withdrawal symptoms, because the duration of withdrawal symptoms was not taken into account. Therefore, withdrawal symptoms should be more comprehensively controlled in future studies. Fifth, a limitation of this study lies in the absence of validated instruments, such as the Pittsburgh Sleep Quality Index (PSQI), for comprehensively assessing sleep quality. Future studies will examine the association of sleep quality and different types of sleep disorders with aggressive behavior.

In conclusion, this investigation demonstrated a significantly higher prevalence of inpatient aggression among alcohol-dependent patients compared to general psychiatric inpatients. In addition to the severity of withdrawal symptoms, key risk factors included shorter sleep duration, elevated NGASR scores and decreased Hb concentrations. Identifying the risk factors associated with aggression among hospitalized patients can help researchers and healthcare professionals develop effective approaches for preventing and managing violent behavior. Additionally, investigating biological influences may enhance our understanding of how social factors are connected to aggressive actions. Future studies will further refine the collection of clinically relevant variables, such as severity of alcohol use, impulsivity of the subjects, type of pharmacotherapy and neuroimaging reports.

## Data Availability

The original contributions presented in the study are included in the article/Supplementary Material. Further inquiries can be directed to the corresponding authors.
